# PCR-Based Serotyping of Streptococcus pneumoniae from Culture-Negative Specimens: Novel Primers for Detection of Serotypes within Serogroup 18

**DOI:** 10.1128/JCM.00419-16

**Published:** 2016-07-25

**Authors:** Arif M. Tanmoy, Senjuti Saha, Gary L. Darmstadt, Cynthia G. Whitney, Samir K. Saha

**Affiliations:** a Child Health Research Foundation, Department of Microbiology, Dhaka Shishu Hospital, Dhaka, Bangladesh; b Department of Molecular Genetics, University of Toronto, Toronto, Canada; c Department of Pediatrics, Stanford University School of Medicine, Stanford, California, USA; d Respiratory Diseases Branch, Centers for Disease Control and Prevention, Atlanta, Georgia, USA; e Bangladesh Institute of Child Health, Department of Microbiology, Dhaka Shishu Hospital, Dhaka, Bangladesh; Boston Children's Hospital

## Abstract

Six multiplex-compatible PCR primers were designed to distinguish Streptococcus pneumoniae serotypes within serogroup 18 from culturable/nonculturable pneumococcal specimens, with no cross-reactivity with other serotypes and respiratory organisms. These primers will aid in the generation of better data on vaccine/nonvaccine serotypes in invasive and carriage pneumococcal surveillance and contribute to future vaccine formulation and impact studies.

## TEXT

Streptococcus pneumoniae is a major cause of infectious disease burden worldwide, especially in children (1). It has a highly diverse polysaccharide capsule that forms the basis of more than 90 different serotypes, whose distribution varies geographically (2, 3). The available pneumococcal conjugate vaccines (PCVs) were designed to provide immunity against the most prevalent invasive serotypes worldwide (3, 4). Understanding the geographical distribution and shifts in prevalence over time of every serotype is important for optimizing vaccine design and understanding postvaccine impact on disease burden.

The current gold standard for serotyping is the Quellung reaction (5). This method is expensive and demands expertise; most importantly, this method cannot discern serotypes in culture-negative specimens (6). This limitation has serious implications in South Asia and Africa, where more than 50% of all meningitis cases are culture negative due to the common practice of antibiotic use prior to seeking care and specimen collection (7, 8). To overcome these limitations, a PCR-based serotyping scheme was optimized to determine serotypes from culture-negative specimens (6). However, the available conventional primers cannot distinguish serotypes within some serogroups, like 18 and 6. Few quantitative PCR (qPCR) schemes are available (9, 10), but they lack completeness (can detect 18B/C only) and were not validated completely due to a lack of culture-negative serogroup-18-positive clinical specimens. This limits our knowledge about serotype distribution and vaccine coverage. This is specifically true for serogroup 18, one of the predominant invasive serogroups worldwide (7, 11–13), which has four different serotypes 18A, 18B, 18C, and 18F. Only serotype 18C has been included in all PCVs (14–16). Distribution data of these serotypes are lacking for all culture-negative cases. Moreover, PCR serotyping directly from respiratory specimens has received attention recently (17–19). Considering the nasopharynx as the key reservoir for transmission (20), the trend of pneumococcus in carriage is crucial for surveying herd immunity and replacement of vaccine serotypes after PCV introduction (21, 22). However, the current PCR serotyping algorithm remains incomplete, as it is unable to detect serotypes within serogroup 18 (23). Although previous attempts to design serotype-specific primers for serogroup 18 were unsuccessful due to the high sequence similarity (23, 24), in this study, we designed new primers to identify and distinguish the serogroup 18 serotypes.

The pneumococcal capsular genes on the capsule polysaccharide (*cps*) locus are flanked by the conserved *dexB* and *aliA* genes (25). The first four genes are highly conserved among all serotypes, but genes in the central part of the locus are more serotype specific and serve as the basis of differentiation between serotypes (23). We designed PCR primers to distinguish serogroup 18 serotypes through manual analysis of a multiple-sequence alignment of all four published *cps* locus sequences (26).

**FIG 1:**
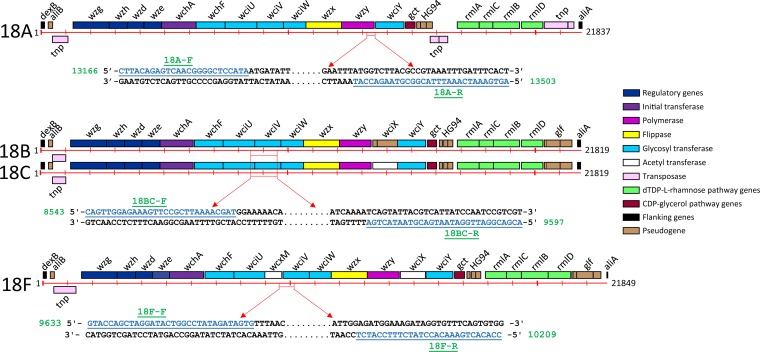
New primers to detect serogroup 18 serotypes (18A, 18B/C, and 18F) and their positions in the *cps* locus. The schematic of the serogroup 18 serotype *cps* locus and organization of the genes with color keys has been adapted from the study by Bentley et al. (26). Genes are represented by boxes and colored according to the gene key, with gene designations indicated above each box. The *cps* loci of 18B and 18C are shown together, as their primers (18BC-F and 18BC-R) were common due to very high sequence identity. The primers used in this study are shown along with their position in the locus (with arrows). Primer sequences are colored and underlined with their designation shown above/below; the genomic position in the *cps* locus is presented beside the primer.

For each selected region of the locus, blastn was used to check its specificity. Once a region was validated to be specific for the serogroup 18 serotypes (i.e., no significant match was found with *cps* loci of other serotypes), primers were designed and checked for physical properties (melting temperature [*T_m_*], G+C% content, hairpins, and dimers) and multiplex PCR compatibility using OligoAnalyzer version 3.1 (Integrated DNA Technologies, USA). Overall, six primers were designed to differentiate between the serogroup 18 serotypes 18A, 18B/C, and 18F (Fig. 1). Primers to distinguish 18B and 18C could not be designed using their CPS sequence due to high (99.99% [21,817/21,819]) sequence identity.

These new primers were evaluated for cross-reactivity with pneumococcal isolates of other serotypes confirmed through Quellung reactions. A library of 167 DNA samples from pneumococcal isolates (pneumococcal DNA library), obtained from invasive and carriage sources, was used to validate these primers. This library contained 67 different serotypes, and 42 isolates belonged to serogroup 18 (11 from 18A, 22 from 18C, and nine from 18F). Further, cross-reaction with other microbial species found in the same niche as S. pneumoniae was appraised using 79 DNA specimens isolated from nasopharyngeal (NP) swabs containing multiple species of bacteria (NP-DNA library). Fifty-four of 79 specimens contained S. pneumoniae strains of 25 different serotypes (one 18A, four 18C, and one 18F), determined by Quellung reactions, along with other bacterial species. Finally, to verify the compatibility with DNA from culture-negative clinical specimens, all new primers were used with 10 culture-negative but serogroup-18-positive clinical specimens (cerebrospinal fluid, *n* = 9; ascitic fluid, *n* = 1); those were confirmed using published sequential multiplex PCR (culture-negative library) (6). Additional information on all DNA samples used here is found in File S1 in the supplemental material.

For 167 pneumococcal isolates, DNA extraction was performed by the boiling method (6); for culture-negative and NP swab specimens, the QIAamp DNA minikit (Qiagen, Germany) was used. For multiplex PCR, two 25-μl reaction mixtures were made, (i) one for the pneumococcal DNA library using 5 μl of FIREPol mastermix (Solis BioDyne, Estonia) and 1 μl of boiled DNA lysate and (ii) one for NP swabs and culture-negative library DNA using 12.5 μl of Qiagen multiplex PCR mastermix (Qiagen, Germany) and 8 μl of DNA. Both reaction mixtures contained 0.4 μM each primer (Eurofins Genomics, USA). Primers (0.4 μM) targeting the *cpsA* locus (23) were added to the mixtures as a positive control. A water-only control was always included. The thermal cycle was 95°C for 15 min, followed by 35 cycles (37 in the case of the culture-negative library to address the challenge of low DNA concentration) of 94°C for 40 s, 61°C for 50 s, and 72°C for 60 s, and then at 72°C for 10 min and held at 4°C. The PCR products were run on a 2% agarose gel.

**FIG 2:**
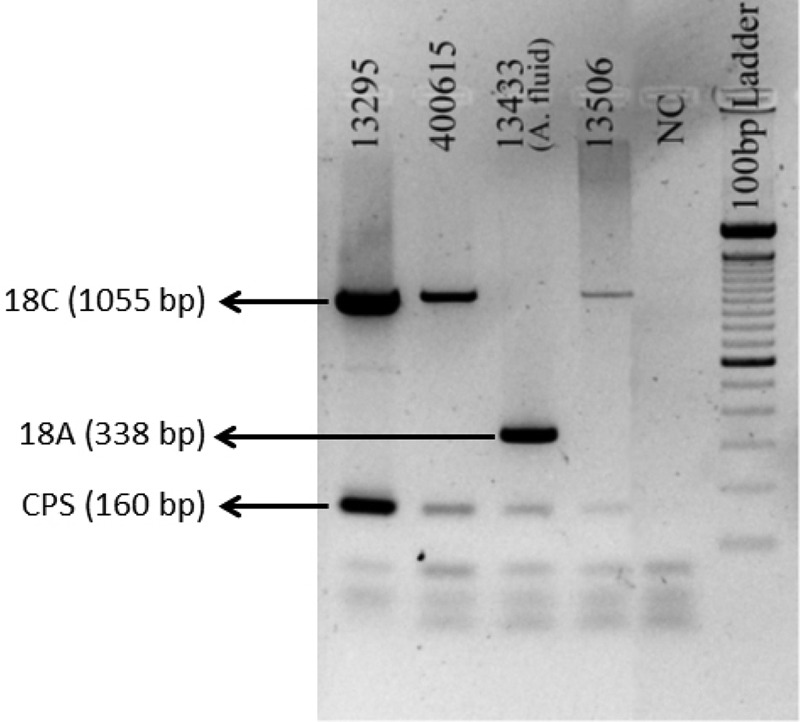
Primer validation with four culture-negative but serogroup-18-positive clinical specimen DNA. Amplified products of multiplex PCR with 18A, 18B/C, and 18F primers, run on 2% agarose gel (at 100 V for 50 min) showed the desired band for 18C (1,055 bp) on all three CSF samples and 18A (338 bp) on the ascitic fluid (A. fluid) specimen. A 100-bp ladder was included in the gel to determine the PCR band size. The gel was stained with SYBR Safe (Invitrogen, USA) and visualized using Gel Doc UV transilluminator (Bio-Rad, USA).

The multiplex PCR on the pneumococcal isolate library with 167 specimens detected all designated isolates of the serogroup 18 serotypes (see Fig. S2 and File S1 in the supplemental material), indicating 100% concordance with the Quellung results. No cross-reaction within and beyond serogroup 18 was observed. All isolates showed positive results for *cpsA* except serogroups 25 and 38, which has been described before (27, 28). For the NP swab library, our multiplex PCR assay was positive only for the pneumococcus-positive NP specimens with specific serotypes 18A/C/F, with no cross-reaction with nonpneumococcal bacterial growth (see Fig. S3 and File S1 in the supplemental material). Most excitingly, our primers amplified DNA in all 10 samples of the culture-negative library, and they revealed 18C in all nine cerebrospinal fluid (CSF) specimens and 18A in the ascitic fluid specimen. Figure 2 shows the PCR products for four samples (additional data in File S1). All reactions showed amplification for *cpsA*, and no dimers with human DNA were seen.

Overall, our results indicate that serotypes within serogroup 18 can be discerned by using the primers described herein, from both culture-positive and culture-negative, invasive and carriage specimens, without any cross-reactivity to non-serogroup-18 serotypes, nonpneumococcal respiratory bacteria, or human DNA. However, they could not be validated with serotype 18B, as none of our DNA libraries from isolates and carriage specimens contained this serotype, implying that our surveillance of multiple modalities did not detect any 18B isolates in the last 2 decades (7, 13, 29, 30). Interestingly, serotype 18B has also not been reported from other countries in this region (31–34). Therefore, we can presume that all 18B/C-positive cases (by our primers) are 18C but simultaneously remain vigilant about the isolation of 18B in this region in the postvaccine era. Recently, an Indian industry, with support from the Bill & Melinda Gates Foundation (BMGF), formulated a PCV without 18C (Keith Klugman, BMGF, personal communication). This is possibly due to the limitation in detecting 18C from culture-negative cases. Therefore, it will be also important to monitor the trend of 18C, using this primer set, in India once the new vaccine is introduced.

In Bangladesh, between 2007 and 2014, serogroup 18 ranked 6th (28/442) among all invasive isolates (7). Quellung-based classification of isolates revealed that 46% of all invasive serogroup 18 strains (13/28) are nonvaccine types 18A and 18F, suggesting the possibility of their emergence as the dominant serotypes post-PCV. Moreover, in recent years, 45% of all serogroup 18 meningitis cases were culture negative, similar to results for other serotypes (7). The lack of isolates from these cases has prevented us from determining the prevalence of specific serotypes and hence limited assessments of the effectiveness of PCVs. The new primers described here will be of paramount significance for comprehensive surveillance on invasive and carriage pneumococcus and PCV effectiveness studies, specifically in South Asian countries, where disease burden is high and prior use of antibiotics is common.

## Supplementary Material

Supplemental material

## References

[1] O'Brien KL, Wolfson LJ, Watt JP, Henkle E, Deloria-Knoll M, McCall N, Lee E, Mulholland K, Levine OS, Cherian T, Hib and Pneumococcal Global Burden of Disease Study Team. 2009 Burden of disease caused by Streptococcus pneumoniae in children younger than 5 years: global estimates. Lancet 374:893–902. doi:10.1016/S0140-6736(09)61204-6. 19748398

[2] Messaoudi M, Milenkov M, Albrich WC, van der Linden MPG, Bénet T, Chou M, Sylla M, Barreto Costa P, Richard N, Klugman KP, Endtz HP, Paranhos-Baccalà G, Telles J-N 2016 The relevance of a novel quantitative assay to detect up to 40 major Streptococcus pneumoniae serotypes directly in clinical nasopharyngeal and blood specimens. PLoS One 11:e0151428. doi:10.1371/journal.pone.0151428. 26986831 PMC4795784

[3] Johnson HL, Deloria-Knoll M, Levine OS, Stoszek SK, Freimanis Hance L, Reithinger R, Muenz LR, O'Brien KL 2010 Systematic evaluation of serotypes causing invasive pneumococcal disease among children under five: the pneumococcal global serotype project. PLoS Med 7:e1000348. doi:10.1371/journal.pmed.1000348. 20957191 PMC2950132

[4] Centers for Disease Control and Prevention. 2015 Epidemiology and prevention of vaccine-preventable diseases: pneumococcal disease. Centers for Disease Control and Prevention, Atlanta, GA http://www.cdc.gov/vaccines/pubs/pinkbook/pneumo.html#vaccines.

[5] Selva L, del Amo E, Brotons P, Muñoz-Almagro C 2012 Rapid and easy identification of capsular serotypes of Streptococcus pneumoniae by use of fragment analysis by automated fluorescence-based capillary electrophoresis. J Clin Microbiol 50:3451–3457. doi:10.1128/JCM.01368-12. 22875895 PMC3486242

[6] Saha SK, Darmstadt GL, Baqui AH, Hossain B, Islam M, Foster D, Al-Emran H, Naheed A, Arifeen SE, Luby SP, Santosham M, Crook D 2008 Identification of serotype in culture negative pneumococcal meningitis using sequential multiplex PCR: implication for surveillance and vaccine design. PLoS One 3:e3576. doi:10.1371/journal.pone.0003576. 18974887 PMC2571985

[7] Saha SK, Hossain B, Islam M, Hasanuzzaman M, Saha S, Hasan M, Darmstadt GL, Chowdury M, El Arifeen S, Baqui AH, Breiman RF, Santosham M, Luby SP, Whitney CG, Pneumococcal Study Group. 2016 Epidemiology of invasive pneumococcal disease in Bangladeshi children before introduction of pneumococcal conjugate vaccine. Pediatr Infect Dis J 35:655–661. 26658530 10.1097/INF.0000000000001037

[8] Moïsi J, Saha S, Falade A, Njanpop-Lafourcade B, Oundo J, Zaidi A, Afroj S, Bakare R, Buss J, Lasi R, Mueller J, Odekanmi AA, Sangare L, Scott JA, Knoll MD, Levine OS, Gessner BD 2009 Enhanced diagnosis of pneumococcal meningitis using the Binax NOW immunochromatographic test of Streptococcus pneumoniae antigen: a multisite study. Clin Infect Dis 48(Suppl 2):S49–S56. doi:10.1086/596481. 19191619 PMC2863072

[9] Tarragó D, Fenoll A, Sánchez-Tatay D, Arroyo L, Muñoz-Almagro C, Esteva C, Hausdorff W, Casal J, Obando I 2008 Identification of pneumococcal serotypes from culture-negative clinical specimens by novel real-time PCR. Clin Microbiol Infect 14:828–834. doi:10.1111/j.1469-0691.2008.02028.x. 18844683

[10] Slinger R, Hyde L, Moldovan I, Chan F, Pernica JM 2014 Direct Streptococcus pneumoniae real-time PCR serotyping from pediatric parapneumonic effusions. BMC Pediatr 14:1. doi:10.1186/1471-2431-14-1. 25060939 PMC4118202

[11] Feikin DR, Klugman KP 2002 Historical changes in pneumococcal serogroup distribution: implications for the era of pneumococcal conjugate vaccines. Clin Infect Dis 35:547–555. doi:10.1086/341896. 12173128

[12] Richter SS, Heilmann KP, Dohrn CL, Riahi F, Diekema DJ, Doern GV 2013 Pneumococcal serotypes before and after introduction of conjugate vaccines, United States, 1999–2011. Emerg Infect Dis 19:1074. doi:10.3201/eid1907.121830. 23763847 PMC3713983

[13] Saha SK, Rikitomi N, Biswas D, Watanabe K, Ruhulamin M, Ahmed K, Hanif M, Matsumoto K, Sack R, Nagatake T 1997 Serotypes of Streptococcus pneumoniae causing invasive childhood infections in Bangladesh, 1992 to 1995. J Clin Microbiol 35:785–787. 9041437 10.1128/jcm.35.3.785-787.1997 PMC229675

[14] Centers for Disease Control and Prevention. 2008 Invasive pneumococcal disease in children 5 years after conjugate vaccine introduction—eight states, 1998–2005. MMWR Morb Mortal Wkly Rep 57:144–148. 18272956

[15] Domingues CMAS, Verani JR, Montenegro Renoiner EI, de Cunto Brandileone MC, Flannery B, de Oliveira LH, Santos JB, de Moraes JC, Brazilian Pneumococcal Conjugate Vaccine Effectiveness Study Group. 2014 Effectiveness of ten-valent pneumococcal conjugate vaccine against invasive pneumococcal disease in Brazil: a matched case-control study. Lancet Respir Med 2:464–471. doi:10.1016/S2213-2600(14)70060-8. 24726406 PMC9003592

[16] Gladstone RA, Jefferies JM, Faust SN, Clarke SC 2012 Pneumococcal 13-valent conjugate vaccine for the prevention of invasive pneumococcal disease in children and adults. Expert Rev Vaccines 11:889–902. doi:10.1586/erv.12.68. 23002969

[17] Wyllie AL, Wijmenga-Monsuur AJ, van Houten MA, Bosch AATM, Groot JA, van Engelsdorp Gastelaars J, Bruin JP, Bogaert D, Rots NY, Sanders EAM, Trzcinski K 2016 Molecular surveillance of nasopharyngeal carriage of Streptococcus pneumoniae in children vaccinated with conjugated polysaccharide pneumococcal vaccines. Sci Rep 6:23809. doi:10.1038/srep23809. 27046258 PMC4820691

[18] van Deursen AM, van den Bergh MR, Sanders EA, Carriage Pilot Study Group. 2016 Carriage of Streptococcus pneumoniae in asymptomatic, community-dwelling elderly in the Netherlands. Vaccine 34:4–6. doi:10.1016/j.vaccine.2015.11.014. 26602269

[19] Hjálmarsdóttir MÁ, Gumundsdóttir PF, Erlendsdóttir H, Kristinsson KG, Haraldsson G 2016 Cocolonization of pneumococcal serotypes in healthy children attending day care centers: molecular versus conventional methods. Pediatr Infect Dis J 35:477–480. doi:10.1097/INF.0000000000001059. 26808723

[20] Simell B, Auranen K, Käyhty H, Goldblatt D, Dagan R, O'Brien KL, Pneumococcal Carriage Group 2012 The fundamental link between pneumococcal carriage and disease. Expert Rev Vaccines 11:841–855. doi:10.1586/erv.12.53. 22913260

[21] Weinberger DM, Malley R, Lipsitch M 2011 Serotype replacement in disease after pneumococcal vaccination. Lancet 378:1962–1973. doi:10.1016/S0140-6736(10)62225-8. 21492929 PMC3256741

[22] Miller E, Andrews NJ, Waight PA, Slack MP, George RC 2011 Herd immunity and serotype replacement 4 years after seven-valent pneumococcal conjugate vaccination in England and Wales: an observational cohort study. Lancet Infect Dis 11:760–768. doi:10.1016/S1473-3099(11)70090-1. 21621466

[23] Pai R, Gertz RE, Beall B 2006 Sequential multiplex PCR approach for determining capsular serotypes of Streptococcus pneumoniae isolates. J Clin Microbiol 44:124–131. doi:10.1128/JCM.44.1.124-131.2006. 16390959 PMC1351965

[24] Morais L, Carvalho Mda G, Roca A, Flannery B, Mandomando I, Soriano-Gabarró M, Sigauque B, Alonso P, Beall B 2007 Sequential multiplex PCR for identifying pneumococcal capsular serotypes from South-Saharan African clinical isolates. J Med Microbiol 56:1181–1184. doi:10.1099/jmm.0.47346-0. 17761480

[25] Paton JC, Morona JK 2000 Streptococcus pneumoniae capsular polysaccharide. American Society for Microbiology, Washington, DC.

[26] Bentley SD, Aanensen DM, Mavroidi A, Saunders D, Rabbinowitsch E, Collins M, Donohoe K, Harris D, Murphy L, Quail MA, Samuel G, Skovsted IC, Kaltoft MS, Barrell B, Reeves PR, Parkhill J, Spratt BG 2006 Genetic analysis of the capsular biosynthetic locus from all 90 pneumococcal serotypes. PLoS Genet 2:e31. doi:10.1371/journal.pgen.0020031. 16532061 PMC1391919

[27] Dias CA, Teixeira LM, Carvalho Mda G, Beall B 2007 Sequential multiplex PCR for determining capsular serotypes of pneumococci recovered from Brazilian children. J Med Microbiol 56:1185–1188. doi:10.1099/jmm.0.47347-0. 17761481

[28] World Health Organization. 2011 Laboratory methods for the diagnosis of meningitis caused by Neisseria meningitidis, Streptococcus pneumoniae, and Haemophilus influenzae: WHO manual, 2nd ed World Health Organization, Geneva, Switzerland http://www.cdc.gov/meningitis/lab-manual/full-manual.pdf.

[29] Saha SK, Rikitomi N, Ruhulamin M, Masaki H, Hanif M, Islam M, Watanabe K, Ahmed K, Matsumoto K, Sack R, Nagatake T 1999 Antimicrobial resistance and serotype distribution of Streptococcus pneumoniae strains causing childhood infections in Bangladesh, 1993 to 1997. J Clin Microbiol 37:798–800. 9986858 10.1128/jcm.37.3.798-800.1999 PMC84560

[30] Saha SK, Baqui AH, Darmstadt GL, Ruhulamin M, Hanif M, El Arifeen S, Santosham M, Oishi K, Nagatake T, Black RE 2003 Comparison of antibiotic resistance and serotype composition of carriage and invasive pneumococci among Bangladeshi children: implications for treatment policy and vaccine formulation. J Clin Microbiol 41:5582–5587. doi:10.1128/JCM.41.12.5582-5587.2003. 14662944 PMC308982

[31] Shah A, Knoll MD, Sharma P, Moisi J, Kulkarni P, Lalitha M, Steinhoff M, Thomas K 2009 Invasive pneumococcal disease in Kanti Children's Hospital, Nepal, as observed by the South Asian Pneumococcal Alliance Network. Clin Infect Dis 48:S123–S128. doi:10.1086/596490. 19191607

[32] Williams EJ, Thorson S, Maskey M, Mahat S, Hamaluba M, Dongol S, Werno AM, Yadav BK, Shah AS, Kelly DF, Adhikari N, Pollard AJ, Murdoch DR 2009 Hospital-based surveillance of invasive pneumococcal disease among young children in urban Nepal. Clin Infect Dis 48:S114–S122. doi:10.1086/596488. 19191606

[33] Jaiswal N, Singh M, Das RR, Jindal I, Agarwal A, Thumburu KK, Kumar A, Chauhan A 2014 Distribution of serotypes, vaccine coverage, and antimicrobial susceptibility pattern of Streptococcus pneumoniae in children living in SAARC countries: a systematic review. PLoS One 9:e108617. doi:10.1371/journal.pone.0108617. 25268974 PMC4182530

[34] Shakoor S, Kabir F, Khowaja AR, Qureshi SM, Jehan F, Qamar F, Whitney CG, Zaidi AK 2014 Pneumococcal serotypes and serogroups causing invasive disease in Pakistan, 2005–2013. PLoS One 9:e98796. doi:10.1371/journal.pone.0098796. 24892937 PMC4043782

