# Supplementary material for: PCR-Based Serotyping of Streptococcus pneumoniae from Culture-Negative Specimens: Novel Primers for Detection of Serotypes within Serogroup 18
Source: J Clin Microbiol. 2016 Jul 25;54(8):2178–81. doi: 10.1128/JCM.00419-16 (PMC4963509; doi:10.1128/JCM.00419-16)
Supplement: Supplemental material [file supp_54_8_2178__index.html]

Supplemental material 

# PCR-Based Serotyping of Streptococcus pneumoniae from Culture-Negative Specimens: Novel Primers for Detection of Serotypes within Serogroup 18

## Supplemental material

- Supplemental file 1 -

  Fig. S2 (Primer validation PCR with pneumococcal DNA from isolates including both invasive and carriage sources)

  PDF, 5.7M
- Supplemental file 2 -

  Fig. S3 (Primer validation PCR with nasopharyngeal DNA samples from nasopharyngeal swab specimens)

  PDF, 3.4M
- Supplemental file 3 -

  Data Set S1 (Data of all DNA specimens used in this study)

  XLSX, 26K
