# Supplementary material for: PCR-Based Serotyping of Streptococcus pneumoniae from Culture-Negative Specimens: Novel Primers for Detection of Serotypes within Serogroup 18
Source: J Clin Microbiol. 2016 Jul 25;54(8):2178–81. doi: 10.1128/JCM.00419-16 (PMC4963509; doi:10.1128/JCM.00419-16)
Supplement: Supplemental material [file JCM.00419-16_zjm999095096so1.pdf]

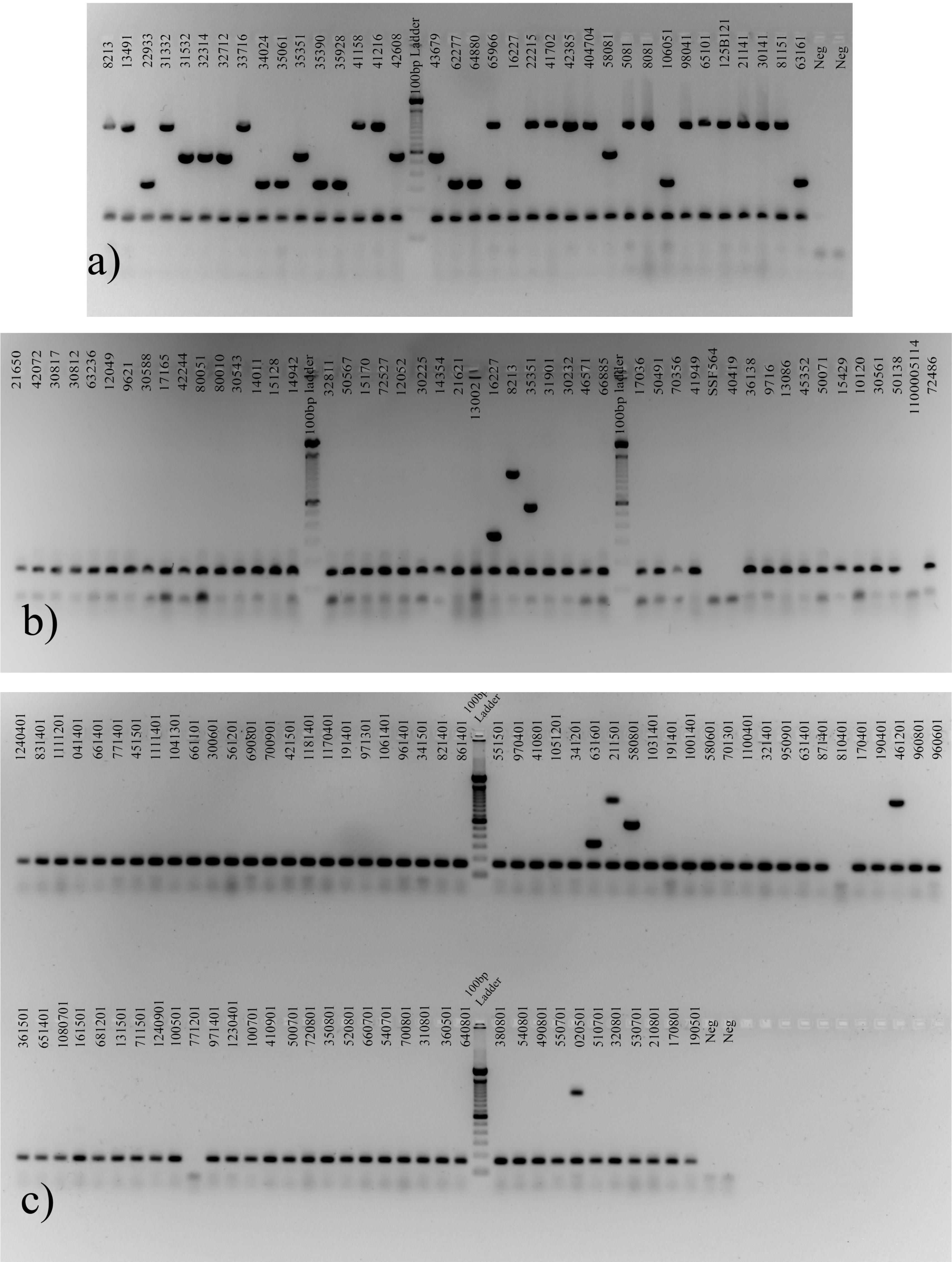

FIG S2. Primer validation PCR with pneumococcal-DNA from isolates including both invasive and carriage sources. Total 167 samples have been run on agarose gels. Detail data of these samples are given in the supplementary file S1. Sample serial no. 1-36 are included in **S2a**, 37-85 in **S2b** and 86-168 in **S2c**. Sample ID of each isolates are mentioned on the gel. One sample (ID- 8213) was run twice in S1a and S1b. Water negative control was included in each gel run. 100bp ladder was run alongside the PCR products to estimate the size. PCR products were run on the gel at 100V for 50 minutes; the gel was stained with SYBR Safe (Invitrogen, USA) and visualized using Gel-Doc UV-trans illuminator (Bio-Rad, USA).
