# Supplementary material for: PCR-Based Serotyping of Streptococcus pneumoniae from Culture-Negative Specimens: Novel Primers for Detection of Serotypes within Serogroup 18
Source: J Clin Microbiol. 2016 Jul 25;54(8):2178–81. doi: 10.1128/JCM.00419-16 (PMC4963509; doi:10.1128/JCM.00419-16)
Supplement: Supplemental material [file JCM.00419-16_zjm999095096so2.pdf]

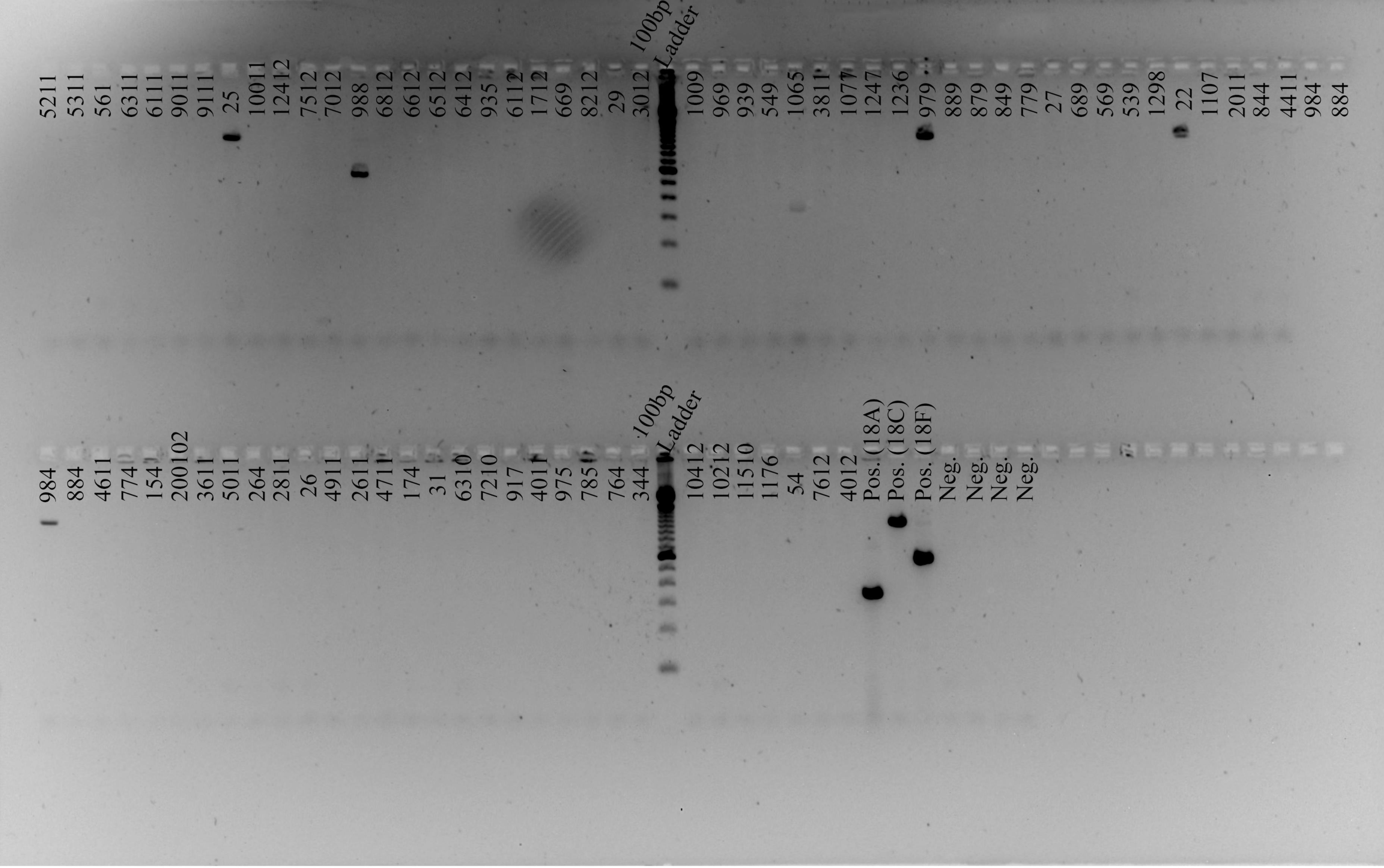

FIG S3. Primer validation PCR with Nasopharyngeal-DNA samples from NP swab specimens. Total 79 samples have been run on agarose gels. Detail data of these samples are given in the supplementary file S1. Sample ID of each isolates are mentioned on the gel. Water negative control was included in each gel run. 100bp ladder was run alongside the PCR products to estimate the size. PCR products were run on the gel at 100V for 50 minutes; the gel was stained with SYBR Safe (Invitrogen, USA) and visualized using Gel-Doc UV-trans illuminator (Bio-Rad, USA)**.**
